# The Burden of Pediatric Encephalitis in the United States

**DOI:** 10.15844/pedneurbriefs-30-10-1

**Published:** 2016-10

**Authors:** Leena B. Mithal

**Affiliations:** 1 Department of Pediatrics, Northwestern University Feinberg School of Medicine, Chicago, IL; and Division of Infectious Diseases, Ann & Robert H. Lurie Children’s Hospital of Chicago, Chicago, IL

**Keywords:** Encephalitis, Epidemiology, Pediatric Critical Care

## Abstract

Investigators from the University of Maryland, University of Colorado, and Arkansas Children’s Hospital studied the epidemiology, management and resource utilization of children admitted to hospitals in the United States with encephalitis.

Investigators from the University of Maryland, University of Colorado, and Arkansas Children’s Hospital studied the epidemiology, management and resource utilization of children admitted to hospitals in the United States with encephalitis. The Pediatric Health Information System database was used to identify children with encephalitis. Data on diagnostic procedures, imaging studies, medications, and outcomes were extracted for the 7298 hospital admissions for encephalitis between 2004 and 2013. The median age at diagnosis was 9 years and length of stay 16 days. Forty percent were admitted to the PICU and these patients had a longer length of stay of 25 days.

Only 60% of children underwent lumbar puncture during their hospitalization, although the authors note that procedures done prior to transfer to the admitting hospital were not captured by the database. Laboratory investigations included cerebrospinal fluid (CSF) and blood cultures, in addition to a variety of viral and arboviral testing. Neuroimaging was obtained in a minority of patients (CT 6%, MRI 9%). Overall, 16.6% were treated with IVIG and 41% received methylprednisolone. Mortality in children with encephalitis was 3%; 81% of patients were confirmed to have been discharged home. The mean charges for hospitalization were $64,604 for acute care floor patients and $260,012 PICU patients. [1]

COMMENTARY. Encephalitis is defined by the presence of an inflammatory process of the brain in association with clinical evidence of neurologic dysfunction [2]. Of pathogens reported to cause encephalitis and in cases with an infectious etiology identified, viruses predominate. However, despite extensive testing, the etiology in the majority of cases remains unknown [3]. Autoimmune causes such as anti-N¬-methyl-D-aspartate receptor encephalitis comprise a significant portion of previously unidentified cases [4]. First line immunotherapy for these conditions may include steroids, IVIG, and plasmapheresis individually or in combination, with early treatment being associated with good outcomes [5]. As discussed by the authors, this large epidemiologic study spans a timeframe during which the recognition and treatment of autoimmune-mediated encephalitides has improved. This study illustrates the significant healthcare burden of pediatric encephalitis, with long durations of inpatient and particularly intensive care hospitalization. Furthermore, prolonged periods of recovery, missed school, and some long term disability are not uncommon.

An etiologic diagnosis should be pursued in cases of childhood encephalitis. In order to identify treatable conditions, improve diagnostic methods, and for prognostic and public health reasons, it is imperative that CSF samples be collected unless contraindicated. The reported rates of herpes simplex virus testing and lumbar puncture were surprisingly low in this study, and these are staples of encephalitis work-up. Neuroimaging is also universally recommended for all patients with encephalitis, with MRI being preferred to CT [2]. Epidemiologic factors including potential exposures should be sought and may provide the only clues to guide clinicians to a specific diagnosis.
